# Emergence of Extensively Drug-Resistant *Salmonella* Typhi Infections Among Travelers to or from Pakistan — United States, 2016–2018

**DOI:** 10.15585/mmwr.mm6801a3

**Published:** 2019-01-11

**Authors:** Kevin Chatham-Stephens, Felicita Medalla, Michael Hughes, Grace D. Appiah, Rachael D. Aubert, Hayat Caidi, Kristina M. Angelo, Allison T. Walker, Noël Hatley, Sofia Masani, June Nash, John Belko, Edward T. Ryan, Eric Mintz, Cindy R. Friedman

**Affiliations:** ^1^Division of Foodborne, Waterborne, and Environmental Diseases, National Center for Emerging and Zoonotic Infectious Diseases, CDC; ^2^Atlanta Research & Education Foundation, Atlanta, Georgia; ^3^Division of Global Migration and Quarantine, National Center for Emerging and Zoonotic Infectious Diseases, CDC; ^4^Public Health — Seattle & King County, Seattle, Washington; ^5^Sacramento County Public Health, Sacramento, California; ^6^Kaiser Permanente, Elk Grove, California; ^7^Division of Infectious Diseases, Massachusetts General Hospital, Boston, Massachusetts; ^8^Department of Immunology and Infectious Diseases, Harvard T.H. Chan School of Public Health, Boston, Massachusetts.

In February 2018, a typhoid fever outbreak caused by *Salmonella enterica* serotype Typhi (Typhi), resistant to chloramphenicol, ampicillin, trimethoprim-sulfamethoxazole, fluoroquinolones, and third-generation cephalosporins, was reported in Pakistan. During November 2016–September 2017, 339 cases of this extensively drug-resistant (XDR) Typhi strain were reported in Pakistan, mostly in Karachi and Hyderabad; one travel-associated case was also reported from the United Kingdom (*1*). More cases have been detected in Karachi and Hyderabad as surveillance efforts have been strengthened, with recent reports increasing the number of cases to 5,372 (*2*). In the United States, in response to the reports from Pakistan, enhanced surveillance identified 29 patients with typhoid fever who had traveled to or from Pakistan during 2016–2018, including five with XDR Typhi. Travelers to areas with endemic disease, such as South Asia, should be vaccinated against typhoid fever before traveling and follow safe food and water practices. Clinicians should be aware that most typhoid fever infections in the United States are fluoroquinolone nonsusceptible and that the XDR Typhi outbreak strain associated with travel to Pakistan is only susceptible to azithromycin and carbapenems.

Typhoid fever is a systemic febrile illness that requires prompt antibiotic treatment.* Worldwide, approximately 12–27 million cases of typhoid fever occur annually (*3*). In the United States, approximately 350 culture-confirmed cases are reported to CDC each year. Most U.S. patients report having traveled internationally within the preceding 30 days. Over the past several decades, the emergence of Typhi that is multidrug resistant (MDR) to historically used first-line antibiotics, such as chloramphenicol, ampicillin, and trimethoprim-sulfamethoxazole, led to the use of fluoroquinolones (e.g., ciprofloxacin) as the first-line treatment (*4*). However, since the early 2000s, increasing fluoroquinolone nonsusceptibility (intermediate or full resistance to ciprofloxacin), especially in South Asia, has led to the use of third-generation cephalosporins (e.g., ceftriaxone) as a recommended first-line treatment.

Local and state health departments report culture-confirmed Typhi to CDC’s National Typhoid and Paratyphoid Fever Surveillance (NTPFS) system (*5*). Information is collected on travel history in the 30 days preceding illness. Public health laboratories in 54 state and local health departments forward all Typhi isolates to CDC’s National Antimicrobial Resistance Monitoring System (NARMS) in batched shipments for antimicrobial susceptibility testing (*6*). The NARMS laboratory uses broth microdilution to determine the minimum inhibitory concentration (MIC) for 14 antimicrobial agents. Resistance is defined by MIC breakpoints established by the Clinical and Laboratory Standards Institute (CLSI) where available (*7*). Typhi isolates are categorized as fluoroquinolone nonsusceptible if their MICs are classified as intermediate (MIC ≥0.12–0.5 *μ*g/mL) or resistant (MIC ≥1.0 *μ*g/mL) to ciprofloxacin. Typhi isolates are defined as MDR if they are resistant to chloramphenicol, ampicillin, and trimethoprim-sulfamethoxazole, and as XDR if they are MDR, nonsusceptible to fluoroquinolones, and resistant to third-generation cephalosporins. In March 2018, CDC enhanced surveillance for typhoid fever by asking state and local health departments to interview every patient with typhoid fever about travel to or from Pakistan and to expedite submission of Typhi isolates from these patients to CDC. Surveillance data from NARMS and NTPFS from 2006–2015 were compared with data from 2016–2018 and reviewed for XDR cases among persons who traveled to Pakistan.

During 2006–2015, a total of 3,538 patients with culture-confirmed typhoid fever were reported to NTPFS (median = 338 patients annually), including 244 (7%) who traveled to only Pakistan in the 30 days before onset (median = 23 patients annually) (Table 1). During 2006–2015, NARMS tested 3,598 Typhi isolates. Among these, 2,350 (65%) were fluoroquinolone nonsusceptible, 418 (12%) were MDR, and none had resistance to ceftriaxone. Fluoroquinolone nonsusceptibility increased from 55% (177 of 323 isolates) in 2006 to 66% (221 of 336) in 2015. Information on international travel was available for 2,242 (62%) patients with isolates tested by NARMS; 169 (8%) traveled to only Pakistan. Of 169 isolates from travelers to Pakistan, 133 (79%) were fluoroquinolone nonsusceptible and 85 (50%) were MDR (Table 1). During 2016–2018, 29 patients with typhoid fever reported travel to or from Pakistan and had isolates tested for antimicrobial susceptibility; among these, five patients had XDR Typhi (Table 2). All patients with XDR Typhi who had traveled to or from Pakistan were children aged 4–12 years and traveled to or from Pakistan during late 2017 through mid-2018.

**TABLE 1:** Number of patients with laboratory-confirmed typhoid fever reported to CDC’s National Typhoid and Paratyphoid Fever Surveillance System, number of isolates tested by the National Antimicrobial Resistance Monitoring System (NARMS), and antibiotic susceptibility — United States, 2006–2015

Characteristic	No.	No. of patients with travel to Pakistan only
**Patients with laboratory-confirmed typhoid fever**	3,538	244
Typhi isolates tested by NARMS*	3,598	169
Fluoroquinolone nonsusceptible (% of isolates tested)^†^	2,350 (65)	133 (79)
MDR (% of isolates tested)^†^	418 (12)	85 (50)
Ceftriaxone-resistant	0	0

**Abbreviation**: MDR = multidrug resistant (resistant to chloramphenicol, ampicillin, and trimethoprim-sulfamethoxazole).

* Representing 2,242 patients with confirmed typhoid fever for whom travel information was available.

^†^ Not mutually exclusive.

**TABLE 2:** Characteristics of 29 patients with culture-confirmed typhoid fever who traveled to or from Pakistan — National Typhoid and Paratyphoid Fever Surveillance System, United States, 2016–2018*

Characteristic	No. (%)
**Sex**	
Male	14 (48)
Female	15 (52)
**Age group (yrs)**	
0–5	5 (17)
6–11	9 (31)
12–17	8 (28)
18–44	6 (21)
45–63	1 (3)
**Traveled to visit friends or relatives**	
Yes	24 (83)
No	1 (3)
Unknown	4 (14)
**Antibiotic resistance^†^**	
Pansusceptible	2 (7)
Fluoroquinolone nonsusceptible	9 (31)
Fluoroquinolone nonsusceptible and MDR	13 (45)
XDR^§^	5 (17)

**Abbreviations:** MDR = multidrug resistant; XDR = extensively drug-resistant.

* Includes patients reported to CDC through October 12, 2018.

^†^ Based on the following four mutually exclusive categories: 1) pansusceptible; 2) fluoroquinolone nonsusceptible; 3) fluoroquinolone nonsusceptible and MDR (resistant to chloramphenicol, ampicillin, and trimethoprim-sulfamethoxazole); and 4) XDR (fluoroquinolone nonsusceptible, MDR, and resistant to third-generation cephalosporins).

^§^ Patients with XDR Typhi were aged 4–12 years.

## Discussion

A large typhoid fever outbreak in Pakistan has resulted in 5,372 XDR Typhi cases reported during 2016–2018, and five travel-related cases in the United States. Approximately 250,000 trips to Pakistan were taken from the United States in 2017 (modeled data from OAG, Inc., https://www.oag.com); travelers to Pakistan might be at risk for acquiring XDR Typhi and having limited treatment options. Spread of the XDR Typhi strain to neighboring countries, such as India, might occur; approximately 2.4 million trips from the United States to India were taken in 2017 (modeled data from OAG, Inc.), and returning travelers from India typically account for 57%–69% of typhoid fever cases reported to CDC (*5*,*8*).

Providers caring for patients with suspected typhoid fever should obtain a travel history, blood and stool cultures, and antimicrobial susceptibility testing. Serologic tests have several limitations and do not yield a bacterial isolate that can be used for antimicrobial susceptibility testing; they should not be used to diagnose typhoid fever. Patients with confirmed typhoid fever should be reported to the local health department. Health departments should notify CDC of typhoid fever cases and send all Typhi isolates to NARMS for antimicrobial susceptibility testing.

Most typhoid fever infections diagnosed in the United States are fluoroquinolone nonsusceptible; therefore, health care providers should not use fluoroquinolones as empiric therapy, especially in returning travelers from South Asia (*8*). Fluoroquinolone nonsusceptibility has been associated with treatment failure or delayed clinical response (*4*). Typhoid fever relapses involving a similar, but often less severe, illness can occur even with appropriate treatment, typically 1–3 weeks after initial clinical improvement (*4*).

The emergence of fluoroquinolone nonsusceptible strains that are resistant to third-generation cephalosporins, such as ceftriaxone, in Pakistan and other countries complicates typhoid fever treatment.^†^ The XDR Typhi strain is only susceptible to azithromycin and carbapenems. Azithromycin should be used to treat patients with suspected uncomplicated typhoid fever who have traveled to or from Pakistan. Azithromycin dosing for typhoid fever is higher than the dosage for more routine indications (*9*). Patients with suspected severe or complicated typhoid fever (which includes encephalopathy, intestinal perforation, peritonitis, intestinal hemorrhage, or bacteremia with sepsis or shock) and who have traveled to or from Pakistan might need to be treated with a carbapenem (*9*). Treatment regimens can be adjusted when culture and sensitivity results are available.

Effective strategies to promote pretravel typhoid vaccination, surveillance with rapid reporting of XDR Typhi cases, and use of alternative empiric treatments when clinical suspicion is high are critical to preventing and treating further travel-associated cases. Two typhoid fever vaccines are available in the United States for travelers: an oral live, attenuated vaccine (Vivotif) and an intramuscular Vi capsular polysaccharide vaccine (Typhim Vi). Both vaccines are moderately effective, protecting 50%–80% of recipients. The oral vaccine can be given to persons aged ≥6 years at least 1 week before travel, and the intramuscular vaccine can be given to persons aged ≥2 years at least 2 weeks before travel (*10*).

The findings in this report are subject to at least two limitations. First, surveillance data from NTPFS and NARMS identify only culture-confirmed infections, which represent a fraction of all infections. Second, some Typhi isolates were from patients for whom a case report form with travel information was not sent to NTPFS; thus travel history and resistance data were not available for all confirmed cases of typhoid fever.

Vaccination and safe food and water practices (only drinking water that is disinfected or bottled and washing hands before eating) while traveling provide the best protection from typhoid fever (*10*). Travelers should seek medical care if they become ill while traveling abroad or after returning home. Early clinical suspicion for typhoid fever can ensure that cultures are sent to the laboratory and that appropriate antibiotic treatment is started quickly, thereby reducing morbidity and mortality. In the United States, collaboration among health care providers, local and state health departments, and CDC is essential to ensuring that emerging resistance patterns are identified quickly and that patients receive appropriate treatment. Globally, public health partners should work to improve prevention efforts that include vaccination in the face of diminishing therapeutic options.

**Summary**

**What is already known about this topic?**

Extensively drug-resistant (XDR) *Salmonella* Typhi causing a typhoid fever outbreak in Pakistan is susceptible only to azithromycin and carbapenems.

**What is added by this report?**

During 2006–2015, 79% of U.S. isolates from typhoid fever patients who traveled to Pakistan were fluoroquinolone nonsusceptible. During 2016–2018, typhoid fever was diagnosed in 29 U.S. patients with recent Pakistan travel; five had XDR Typhi.

**What are the implications for public health practice?**

Vaccination can help prevent typhoid fever. Fluoroquinolones should not be used for empiric treatment of typhoid fever patients who traveled to South Asia. Patients with travel to Pakistan should be treated with azithromycin for uncomplicated typhoid fever and with carbapenems for complicated disease.

## Footnotes

* https://www.cdc.gov/typhoid-fever/symptoms.html.

† https://doi.org/10.1128/mBio.02112-18.
